# Lumen Apposing Metal Stents for Gastrojejunal Anastomotic Stricture Following Metabolic Bariatric Surgery

**DOI:** 10.1007/s11695-025-07891-9

**Published:** 2025-06-10

**Authors:** Preekesh Suresh Patel, Samuel Reddish, Andrew Maurice, Jason Robertson, Michael Booth, Marius van Rijnsoever

**Affiliations:** 1 Te Whatu Ora - Waitemata, Auckland, New Zealand; 2 University of Auckland, Auckland, New Zealand

**Keywords:** Gastric bypass, Self expandable metallic stents, Stenosis, Bariatric surgery, Dilatation

## Abstract

**Background:**

The standard of care for gastrojejunal anastomotic stricture following metabolic bariatric bypass surgery is endoscopic balloon dilatation, with revisional surgery as a last line option. The use of lumen-apposing metal stents is expanding to include many gastrointestinal benign and malignant causes in selected cases. They may provide an additional treatment option for post-bypass strictures.

**Methods:**

A single centre, retrospective outcomes analysis was performed over a 3-year period of patients with gastrojejunal anastomotic stricture following metabolic bariatric surgery that was treated with a lumen-apposing metal stent. Primary outcomes assessed were clinical success and perforation. Multiple secondary outcomes were assessed regarding safety and endoscopy use.

**Results:**

Of 88 patients that had lumen-apposing metal stents placed, 20 satisfied selection criteria. Eleven patients (55%) had at least one balloon dilatation prior to stent placement. All patients achieved clinical success 20 (100%) with zero perforations. Technical success was achieved in 19 patients (95%). There were four (20%) recurrent strictures, two stent migrations (10%) (with no associated complication), and one in-stent food bolus obstruction (5%). One patient ultimately required surgical revision (5%). Three patients had endoscopy within 30 days of stent removal (15%), and five patients required unplanned endoscopy with the stent in situ (25%).

**Conclusion:**

Lumen-apposing metal stents within our study show potential as another treatment option for gastrojejunal anastomotic stricture following gastric bypass.

**Graphical Abstract:**

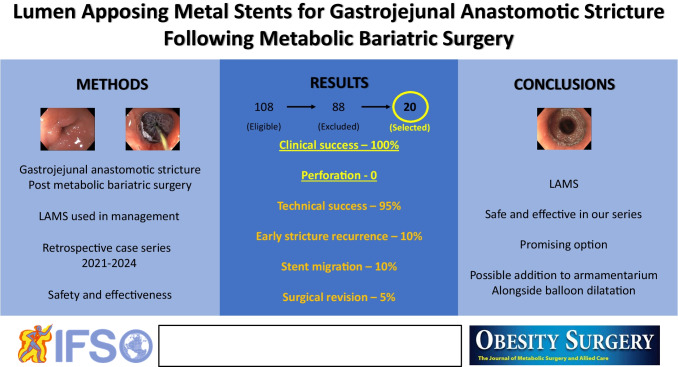

**Supplementary Information:**

The online version contains supplementary material available at 10.1007/s11695-025-07891-9.

## Introduction

Gastrojejunal anastomotic stricture (GJAS) rates following metabolic bariatric surgery (MBS) range from 0.3 to 30% [1, 2]. Endoscopic balloon dilatation (BD) and revisional surgery are established treatment modalities. BD is effective, repeatable and allows same day discharge [3]. There is risk of perforation, bleeding and recurrence. Revisional surgery allows construction of a wider anastomosis and the option of concurrent antrectomy (recurrence risk reduction) at the cost of the increased risks of revisional surgery. Lumen apposing metal stents (LAMS) are becoming increasingly utilised for upper gastrointestinal tract disorders such as the complications of cholelithiasis (in conventional and surgically altered anatomy), benign strictures following gastric bypass and palliative bypass for malignant biliary and gastric outlet obstruction [4]. LAMS are potentially a useful additional option for GJAS post-MBS. Their design inherently reduces migration risk (dumbbell shape), perforation risk (progressive radial expansion) and limits the sequelae of perforation (covered nature) [4]. They may also reduce the total number of endoscopies required and mitigate the requirement for revisional surgery. The main limitations of LAMS include the risks of stent migration/obstruction, perforation and cost [4]. We aim to define our local LAMS outcomes in the management of GJAS post-MBS to determine if LAMS could be a suitable additional option to the current standard of care.

## Methods

### Setting

A retrospective outcome analysis was carried out at a single bariatric unit in Waitemata, New Zealand. Institutional review board approval was obtained. All patients with LAMS inserted between May 2021 and May 2024 were identified using the local stent register.

### Selection Criteria

**Fig. 1:**
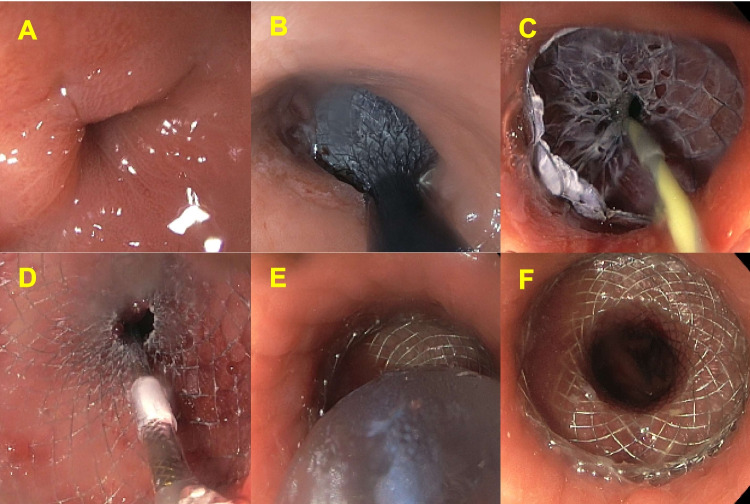
**A** Gastrojejunal anastomotic stricture. **B**, **C** and **D**) After traversing the gastrojejunal anastomotic stricture with a soft tip jag wire under fluoroscopic guidance, the LAMS system (AXIOS or SPAXUS) is deployed (first the distal flange and then the proximal flange) over the wire, and the introducer is removed. **E** Gentle balloon dilatation of the LAMS following insertion is performed to augment early expansion. **F** The deployed and dilated LAMS in situ, with the stricture treated and the Roux limb visible through the stent.

Inclusion criteria were: previous bariatric gastric bypass and GJAS (inability to intubate the roux limb with a standard 10-mm gastroscope (Fig. 1A)), managed with a LAMS. GJAS were defined as early (< 4 5-days post-operative) and late (≥ 45-days post-operative) [1]. Only the first LAMS was counted if multiple was used for the same patient. Exclusion criteria were: previous non-bariatric bypass and GJAS secondary to perforation or silastic rings.

### LAMS insertion

LAMS were deployed under proceduralist sedation or general anaesthesia (combined endoscopist/anaesthetist decision), and the technique is summarised in Fig. 1.

### Outcomes

The primary effectiveness outcome was clinical success (tolerance of two bariatric fluid meals within 24 hours). The primary safety outcome was perforation (diagnosed endoscopically, radiologically and/or surgically). Secondary outcomes included technical success (satisfactory LAMS placement at first attempt, as per treating endoscopist), stricture recurrence within 45 days, stent migration, stent associated bowel obstruction, in-stent obstruction, surgical revision (within 1-year), endoscopy within 30-days of LAMS removal and unplanned endoscopy with LAMS in situ.

## Results

One hundred eight patients had LAMS placed within the study period. Eighty-eight patients were excluded due to: previous non-bariatric bypass and different indications (endoscopic pancreatic necrosectomy, cyst drainage and stenosis associated with silastic rings or perforation). Twenty patients were included. Baseline characteristics are summarised in Table 1. The cohort was predominantly female, NZ European, had previous RYGB and had BD prior to LAMS insertion.

**Table 1:** **Baseline characteristics**

	(*n* = 20)
**Mean age (years)** ± ***SD* [range]**	47.3 ± 14.1 (23–74)
**Gender**	
Female	17 (85%)
Male	3 (15%)
**Ethnicity**	
NZ European	18 (90%)
Maori	1 (5%)
Samoan	1 (5%)
**Bypass type**	
RYGB	19 (95%)
OAGB	1 (5%)
**Gastrojejunal anastomotic stricture**	
Early	8 (40%)
Late	12 (60%)
**Prior balloon dilatation**	
0	9 (45%)
1	7 (35%)
2	4 (20%)*
**Anastomosis size at time of index surgery**	
32 F	7 (35%)
36 F	12 (60%)
Unknown	1 (5%)^#^

Findings displayed as *N* (%) unless otherwise stated. Anastomosis size expressed in French size (1 mm = 3 F)

*SD* standard deviation, *NZ* New Zealand, *RYGB* Roux-en-Y gastric bypass, *OAGB* one anastomosis gastric bypass

*Of these patients, the total numbers of balloon dilatation prior to LAMS insertion were two, two, four and six)

^#^Unknown as procedure was performed overseas, and operation note was not accessible

**Fig. 2:**
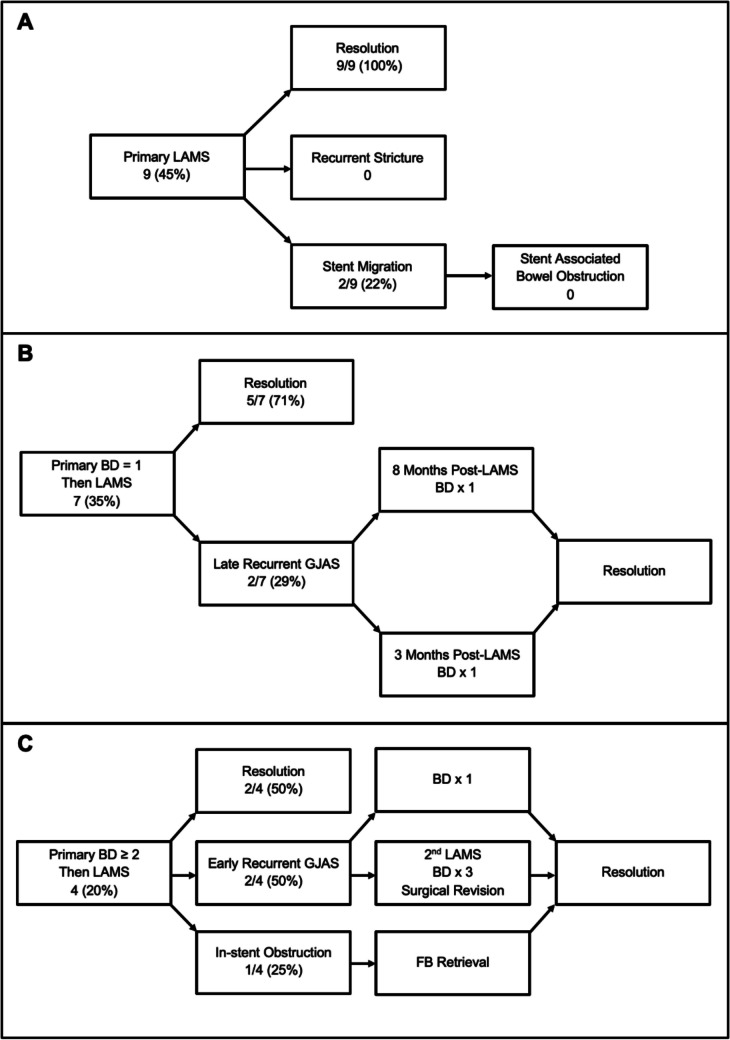
Case resolutions and complications by index stricture intervention. A) Index intervention was LAMS. B) Index intervention was one balloon dilatation. C) Index intervention was ≥ 2 balloon dilatations. LAMS – lumen apposing metal stents, BD – balloon dilatation, FB – food bolus

The results are summarised in Table 2. The single technical failure (misdeployment) was rectified immediately. There were two early recurrent GJAS with their management outlined in Fig. 2. One of these patients had a second LAMS for 3 months and a further three BD prior to surgical revision. There were two delayed recurrent strictures (3 months and 8 months) both managed successfully with a single BD. Both stent migrations were uncomplicated and identified at planned endoscopic removal. The indication for post-LAMS removal endoscopy within 45 days was food intolerance (one was normal; two were recurrent GJAS). The indications for unplanned endoscopy with LAMS in situ were vomiting (normal), to secure the LAMS, in-stent obstruction and feeding tube insertion.

**Table 2:** **Primary and secondary outcomes**

	(*n* = 20)
**Primary effectiveness outcome**	
Clinical success	20 (100%)
**Primary safety outcome**	
Perforation	0
**Secondary outcomes**	
Technical success	19 (95%)
Early stricture recurrence	2 (10%)
Late stricture recurrence	2 (10%)
Stent migration	2 (10%)
Stent associated bowel obstruction	0
In-stent obstruction	1 (5%)
Surgical revision	1 (5%)
Endoscopy within 30-days of LAMS removal	3 (15%)
Unplanned endoscopy with LAMS in situ	5 (25%)

Findings displayed as *N* (%) unless otherwise stated

*LAMS* lumen apposing metal stent

## Discussion

The results demonstrate 100% clinical success, no perforations, 95% technical success and favourable complication rates for GJAS management post-MBS using LAMS. These findings are important as they add to the existing literature, demonstrating the potential of LAMS as an adjunctive treatment option in benign stricture management [5–9].

Reported clinical success rates range widely between 82.6 and 98.4%, reflecting variability in definitions [5–7]. Our rate is likely influenced by low participant numbers and our definition of clinical success being reflective of early symptomatic relief. Our rate of complete resolution without further intervention was 80% which is a more meaningful consideration in stricture management. Our technical success rate of 95% correlates to reported rates, all likely impacted by endoscopists being specifically trained in interventional bariatric endoscopy [5–7].

Safety is paramount when considering LAMS suitability for the off-label indication of benign strictures. LAMS delivering slow, progressive dilatation likely contributed to our cohorts zero perforation rate. BD is associated with a 2.8% risk of perforation per dilatation and 10% perforation risk overall [1, 2]. Fibrosed ischaemia-related GJAS are relatively resistant to BD, leading to recurrent BD and increased perforation risk [2]. In the event of perforation, LAMS is beneficial as it provides an instant seal. Some of our cohorts may have had ischaemia-related GJAS, including the single patient that underwent four BD and two LAMS prior to surgical revision.

Our cohorts’ 10% stent migration rate is within the reported range (8–30%) [5–7]. We recommend abdominal X-ray prior to scheduled LAMS removal to prevent unnecessary endoscopy for migrated stents. The single episode of in-stent food bolus obstruction we report highlights the importance of patient education for stent diets to prevent aspiration and limit unnecessary endoscopy/admission.

There are no established guidelines on the management of GJAS, and the role of LAMS is not clearly defined. Our rate of GJAS resolution without further intervention dropped as the number of BD before LAMS increased. Decision-making regarding LAMS use for GJAS should consider stricture aetiology and number of prior BD [2]. Beyond this, surgical revision may be required. After two BD, LAMS has demonstrated cost-effectiveness for benign post-surgical upper gut strictures [10]**.** Unplanned endoscopies should also be given consideration, with eight being required in our series.

Limitations of our study include its retrospective nature, low case numbers and a paucity of information regarding the rationale for timing of LAMS use. Including all local GJAS cases (with or without LAMS use) would have provided a useful standard of care group. The findings of this study show that LAMS shows potential in the field of benign GJAS management and should be explored further. Future studies should be prospective, multicentre, double-armed, consider timing of LAMS implementation and perform a cost analysis.

## Conclusion

In summary, within our study setting, LAMS are a promising treatment consideration alongside BD for the management of GJAS following MBS. They could increase the available treatment options and contribute to benign stricture management guidelines.

## Supplementary Information

Below is the link to the electronic supplementary material.

Supplementary file1 (DOCX 419 KB)

## Data Availability

No datasets were generated or analysed during the current study.
