# Supplementary material for: Lumen Apposing Metal Stents for Gastrojejunal Anastomotic Stricture Following Metabolic Bariatric Surgery
Source: Obes Surg. 2025 Jun 10;35(7):2755–61. doi: 10.1007/s11695-025-07891-9 (PMC12271261; doi:10.1007/s11695-025-07891-9)
Supplement: Supplementary file 1 — Supplementary file1 (DOCX 419 KB) [file 11695_2025_7891_MOESM1_ESM.docx]

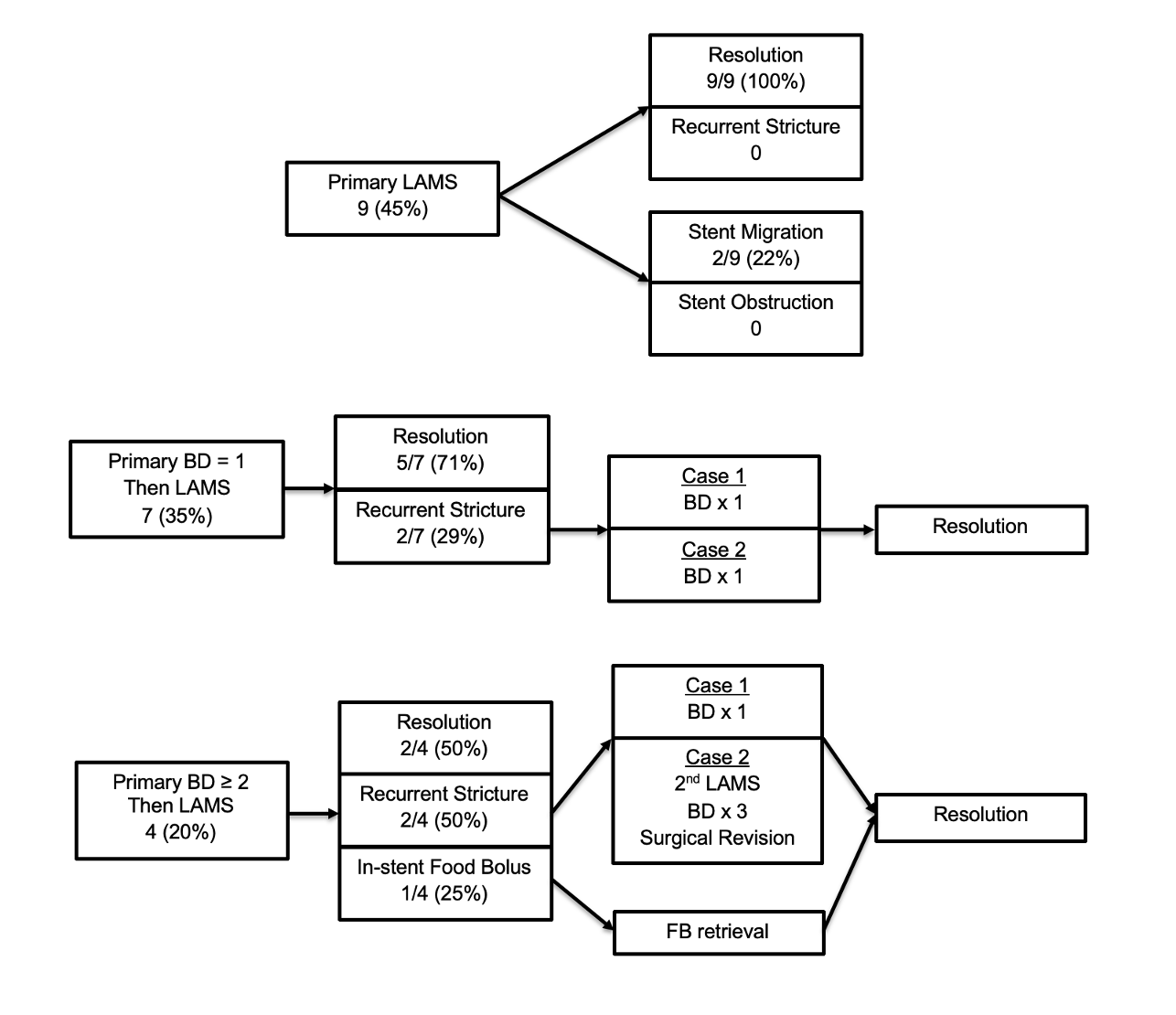


BD x 1

Resolution

2/4 (50%)

2^nd^ LAMS

BD x 3

Surgical Revision

Resolution

Early Recurrent GJAS

2/4 (50%)

Primary BD ≥ 2

Then LAMS

4 (20%)

FB Retrieval

In-stent Obstruction

1/4 (25%)

Resolution

3 Months Post-LAMS

BD x 1

Resolution

8 Months Post-LAMS

BD x 1

Late Recurrent GJAS

2/7 (29%)

Resolution

5/7 (71%)

Primary BD = 1

Then LAMS

7 (35%)

Primary BD ≥ 2

Then LAMS

4 (20%)

Early Recurrent GJAS

2/4 (50%)

Resolution

2/4 (50%)

In-stent Obstruction

1/4 (25%)

BD x 1

2^nd^ LAMS

BD x 3

Surgical Revision

Resolution

FB Retrieval

Primary BD ≥ 2

Then LAMS

4 (20%)

Early Recurrent GJAS

2/4 (50%)

Resolution

2/4 (50%)

In-stent Obstruction

1/4 (25%)

BD x 1

2^nd^ LAMS

BD x 3

Surgical Revision

Resolution

FB Retrieval

Primary BD ≥ 2

Then LAMS

4 (20%)

Early Recurrent GJAS

2/4 (50%)

Resolution

2/4 (50%)

In-stent Obstruction

1/4 (25%)

BD x 1

2^nd^ LAMS

BD x 3

Surgical Revision

Resolution

FB Retrieval

Primary BD ≥ 2

Then LAMS

4 (20%)

Early Recurrent GJAS

2/4 (50%)

Resolution

2/4 (50%)

In-stent Obstruction

1/4 (25%)

BD x 1

2^nd^ LAMS

BD x 3

Surgical Revision

Resolution

FB Retrieval

Primary BD ≥ 2

Then LAMS

4 (20%)

Early Recurrent GJAS

2/4 (50%)

Resolution

2/4 (50%)

In-stent Obstruction

1/4 (25%)

BD x 1

2^nd^ LAMS

BD x 3

Surgical Revision

Resolution

FB Retrieval

Primary BD ≥ 2

Then LAMS

4 (20%)

Early Recurrent GJAS

2/4 (50%)

Resolution

2/4 (50%)

In-stent Obstruction

1/4 (25%)

BD x 1

2^nd^ LAMS

BD x 3

Surgical Revision

Resolution

FB Retrieval

Primary BD ≥ 2

Then LAMS

4 (20%)

Early Recurrent GJAS

2/4 (50%)

Resolution

2/4 (50%)

In-stent Obstruction

1/4 (25%)

BD x 1

2^nd^ LAMS

BD x 3

Surgical Revision

Resolution

FB Retrieval

Primary BD ≥ 2

Then LAMS

4 (20%)

Early Recurrent GJAS

2/4 (50%)

Resolution

2/4 (50%)

In-stent Obstruction

1/4 (25%)

BD x 1

2^nd^ LAMS

BD x 3

Surgical Revision

Resolution

FB Retrieval

Resolution

9/9 (100%)

Stent Associated Bowel Obstruction

0

Recurrent Stricture

0

Primary LAMS

9 (45%)

Stent Migration

2/9 (22%)
